# Anxiety and depression among healthcare workers during COVID-19 pandemic: A cross-sectional study

**DOI:** 10.1016/j.heliyon.2021.e08570

**Published:** 2021-12-08

**Authors:** Saeideh Motahedi, Nasrin Fadaee Aghdam, Mahboobeh Khajeh, Robabe Baha, Roqayeh Aliyari, Hossein Bagheri, Abbas mardani

**Affiliations:** a Student Research Committee, School of Nursing and Midwifery, Shahroud University of Medical Sciences, Shahroud, Iran; b School of Nursing and Midwifery, Shahroud University of Medical Sciences, Shahroud, Iran; c Tehran University of Medical Science, Tehran, Iran; d Ophthalmic Epidemiology Research Center, Shahroud University of Medical Sciences, Shahroud, Iran; e Nursing Care Research Center, Department of Medical Surgical Nursing, School of Nursing and Midwifery, Iran University of Medical Sciences, Tehran, Iran

**Keywords:** COVID-19, Anxiety, Depression, Healthcare workers

## Abstract

**Introduction:**

During a pandemic, healthcare workers (HCWs) are exposed to many stresses that predispose them to psychological disorders. This study aimed to evaluate the impact of the coronavirus disease 2019 (COVID-19) pandemic on the anxiety and depression level of HCWs and determine the relationship between them in terms of their demographic characteristics.

**Material and methods:**

This study used a cross-sectional design. The participants consisted of clinical, administrative, and cleaning staff who were working in a referral COVID-19 hospital in an urban area of Iran. The census sampling method was used for recruiting the participants from May to August 2020. The Generalized Anxiety Disorder-7 (GAD-7) questionnaire and the Center for Epidemiologic Studies Depression (CES-D) Scale were employed to collect data. Then, data were analyzed using multivariable linear regression analysis.

**Results:**

One hundred forty HCWs participated in this study. The mean scores of anxiety and depression were 6.64 (4.86) and 18.21 (10.59), respectively. There was a significant direct association between anxiety and depression (P < 0.001). In addition, female gender (P = 0.01) and having a history of infection with COVID-19 (P = 0.001) were associated with a higher level of anxiety. Moreover, having a history of being quarantined due to COVID-19 was associated with a higher level of depression (P = 0.03).

**Conclusion:**

According to the findings of the present study, considering the mental health of HCWs during the generalized anxiety outbreak of COVID-19 should be a priority, and appropriate interventions should be planned to improve their psychological condition.

## 1 Introduction

The outbreak of unknown pneumonia in late 2019 in China led to the introduction of a new type of coronavirus, causing a new respiratory disease. With the rapid spread of the disease in China and other countries, the new coronavirus, scientifically known as severe acute respiratory syndrome coronavirus 2 (SARS-CoV-2) and the resulting disease called Coronavirus Disease 2019 (COVID-19), aroused great concern among the people all around the world [1]. The World Health Organization (WHO) stated that the virus outbreak was the cause of a public health emergency worldwide [2]. According to the latest statistics declared by the WHO, approximately 226 million confirmed cases of COVID-19 have been identified all over the world, and more than 4654000 people have lost their lives until 16 September 2021 [3]. Meanwhile, Iran is one of the countries with the highest morbidity and mortality rates. Five waves of COVID-19 disease have occurred since the pandemic outbreak in Iran. According to official reports, more than 5 million cases and nearly 116 thousand deaths resulting from COVID-19 have been reported in Iran until 16 September 2021 [4].

People with COVID-19 report a wide range of mild to severe symptoms. These symptoms may appear two to 14 days after exposure to the virus [5] which include fever, cough, sputum production, shortness of breath, dyspnea, anorexia, and muscle pain [6]. Regardless of the symptoms, the COVID-19 pandemic has broadly increased anxiety and changed individual behaviors due to its infectious nature [7]. Most individuals are exposed to an unprecedented stressful situation for an unknown period, which may increase stress, anxiety, and depression levels, as well as disrupt sleep [8]. Stanton et al. found that negative changes in physical activity and sleep, smoking, and alcohol intake were associated with higher levels of depression, anxiety, and stress symptoms in 1491 adults since the onset of COVID-19 in Australia [9].

Everyone in the community is exposed to the adverse effects of the COVID-19, but more attention should be paid to the healthcare workers (HCWs) because they are at the forefront of the fight against this disease and play a vital role in the healthcare system [10, 11]. In addition to annoying exhausting work conditions and occupational hazards, direct contact with COVID-19 patients puts HCWs at greater risk of disease exposure [11, 12]. Therefore, pandemic preparedness is important, especially for non-medical staff who may not be appropriately familiar with the concepts of infectious diseases [12].

According to statistics presented by the WHO, “while health workers represent less than 3% of the population in the large majority of countries and less than 2% in almost all low- and middle-income countries, nearly 14% of COVID-19 cases reported to the WHO are among health workers” [13]. According to other reports, more than 10% of confirmed COVID-19 cases are HCWs, and Iranian health officials stated that more than 12,000 HCWs have been infected, 164 of whom passed away. This high rate of infection had a tremendous impact on the healthcare system [14].

Direct contact of HCWs with patients puts them at high risk of infection, increasing their fear and anxiety [15, 16]. COVID-19 can be an independent risk factor for stress among HCWs [17]. Chen et al.‘s study found that 16.63% of HCWs reported symptoms of moderate/severe anxiety, 18.29% had moderate/severe depression, and 24.50% of them experienced moderate/severe anxiety and depression simultaneously during the COVID-19 outbreak in China [18].

Mental health disorders were also observed during the severe acute respiratory syndrome (SARS) outbreak. Previous studies demonstrated that post-traumatic stress disorder (PTSD) and depression disorders were common among HCWs during the SARS outbreak [19, 20, 21]. Overall, a systematic review concluded that diseases such as SARS, Middle East respiratory syndrome (MERS), and COVID-19 had a substantial impact on the HCWs’ mental health so that they experienced fear, insomnia, psychological distress, burnout, anxiety features, depression symptoms, and PTSD features [22].

It is important to examine the mental health problems such as anxiety and depression in the HCWs because maintaining their mental and physical health is an important factor in providing high-quality healthcare. Therefore, this study aimed to evaluate the impact of the COVID-19 pandemic on the anxiety and depression level of HCWs and examine the relationship between them concerning their demographic characteristics.

## 2 Materials and methods

### 2.1 Study design and setting

A cross-sectional design was applied in this study. The study setting was a referral hospital in an urban area of Iran that provided care for patients diagnosed with COVID-19.

### 2.2 Participants and sampling

All clinical, administrative, and cleaning staff who were working in the hospital for at least one month during the COVID-19 pandemic were eligible to participate in the study. The exclusion criteria were participating in any psychological counseling program in the past two weeks and experiencing a stressful event in the past month such as the death of a loved one or the infection of a first-degree relative with COVID-19.

According to a similar study [23], the prevalence of severe depression and anxiety was estimated at 0.24. It was assumed that the distribution of depression and anxiety is normal. Therefore, the required sample size was calculated to be 143 participants based on the Sample size formula with alpha, beta, and d values of 0.05, 0.2, and 0.1, respectively as Eq. (1).

(1) $$n=\frac{p\left(1-p\right){\left(z_{1}-\alpha /2+z_{1}-\beta \right)}^{2}}{d^{2}}=\frac{0.24\left(0.76\right){\left(1.96+0.84\right)}^{2}}{0.1^{2}}=143$$

The census method sampling was used to recruit the eligible participants. First, the researchers referred to the hospital wards and then used the face-to-face conversation to inform HCWs and evaluate their criteria. HCWs responded to the researchers if they were willing.

### 2.3 Study instruments

Three self-administered questionnaires consisting of a demographic questionnaire, the Generalized Anxiety Disorder-7 (GAD-7) questionnaire, and the Center for Epidemiologic Studies Depression (CES-D) scale were used for data collection.

A demographic questionnaire was used to collect the demographic data. The questionnaire included questions about age, gender, marital status, types of employees, education level, type of work shift, work unit, work experience, working hours per week, number of days work during the COVID-19 pandemic, history of infection with COVID-19, and history of being quarantined due to COVID-19 (Appendix A).

The GAD-7 questionnaire was employed to evaluate anxiety in the participants (Appendix B). The initial version of the GAD questionnaire, which had 13 items, was evaluated on 2740 adult patients between 2004 and 2005 in 15 primary care clinics in the United States, and finally, 7 items were approved to be included in the final version [24]. The results obtained in the initial study revealed the acceptable validity and reliability of this tool. This scale consists of 7 items that evaluate a person's feelings and behaviors in the last two weeks using a 4-point Likert scale ranging from 0 “no time” to 3 “almost every day”. The score of the tool ranges between 0 and 21 in which a score of ten or higher indicates a GAD [24, 25]. The psychometric properties of this tool were confirmed for the Iranian people, and its internal consistency (Cronbach's alpha coefficient) was reported to be 0.876 [26].

The CES-D scale was also used to collect data regarding participants' depression status (Appendix C). This tool consists of 20 items that express a person's feelings of depression in the last two weeks. Each item is on a 4-point Likert scale ranging from 0 “rarely or none of the time” to 3 “most or almost all the time”. The total score on the scale ranged from 0 to 60 in which a high score indicates greater depression symptoms. Accordingly, a score less than 15 and a score of 15–21 characterized normal (i.e., no depression) and mild to moderate depression, respectively, and a score equal to or greater than 21 showed severe depression [27]. Cronbach's alpha coefficient of the Persian version of this tool was reported as greater than 0.70, indicating high internal consistency [28].

### 2.4 Data collection

**Figure 1:**
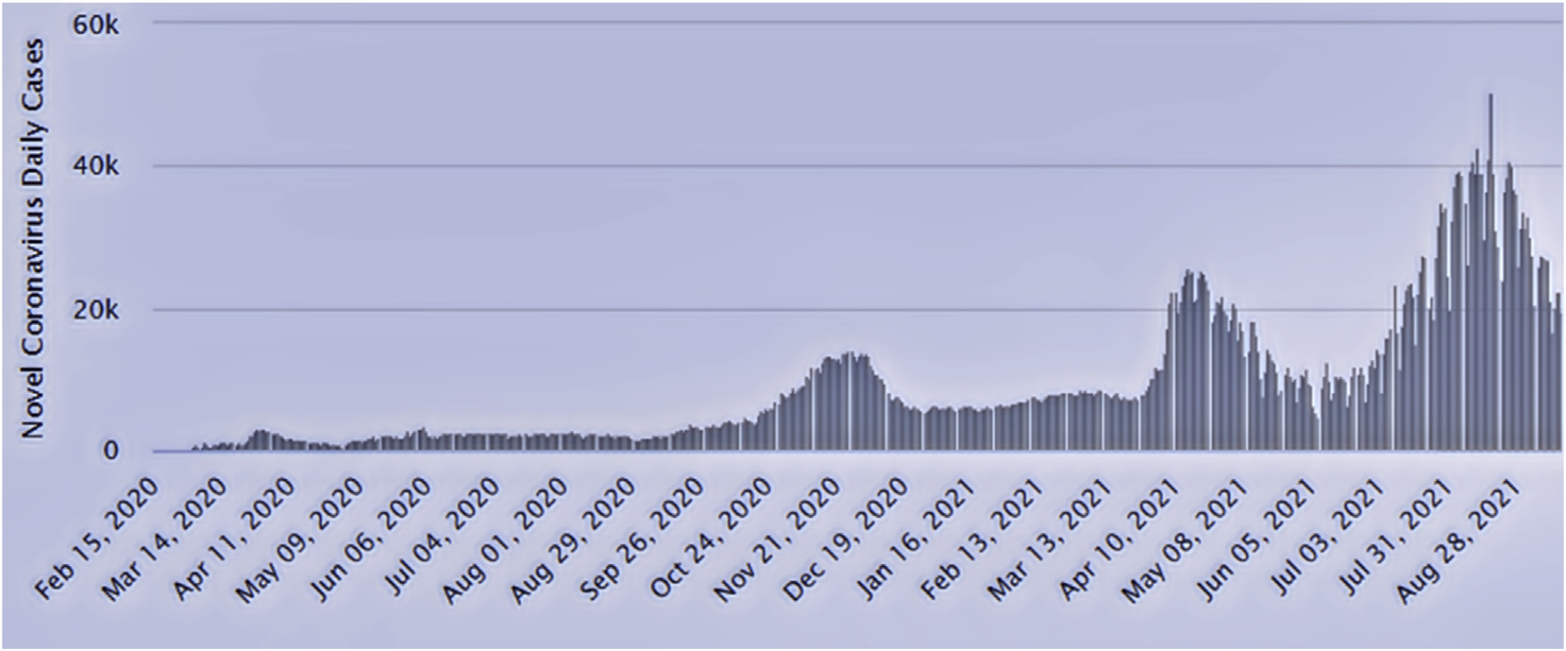
Daily Spread Trends of COVID-19 in Iran. *Note*. COVID-19: Coronavirus disease 19.

Figure 1 presents the daily spreading trends of COVID-19 in Iran [29]. Data were collected between the end of the first wave (May 2020) to the beginning of the third wave (August 2020) in Iran. After referring to the study setting in the different work shifts (morning, evening, and night), the eligible participants were identified. Adhering to the principles of personal protection, the researchers met with the eligible participants during the break between work and explained the study purpose. Then, the study questionnaires were provided to the participants in paper form with sufficient explanations on how to complete the questionnaires. The time required to complete the questionnaire was about 15 min. The participants were given options to complete questionnaires during the same shift work or until the next two weeks to alleviate the effect of fatigue on responses. Two reminders were sent to those who had not returned the questionnaires using mobile text messages.

### 2.5 Ethical considerations

The research proposal was approved by the Research Ethics Committee of Shahroud University of Medical Sciences (IR.SHMU.REC.1399.046). Sufficient information regarding the aims of the study was given to the potential participants, and they were assured of the confidentiality of the collected data. Oral and written informed consent was obtained from the willing participants. The willing participants signed the informed consent form which contained all the required information about the study.

### 2.6 Data analysis

The information recorded in the questionnaire was fed into SPSS software, version 25. Descriptive statistics including the mean, standard deviation (SD), as well as frequency (percentage) were administered for continuous and categorical variables to summarize the data, respectively. Multivariable linear regression analysis was conducted to investigate the association among demographic characteristics, depression, and anxiety. The significance level was set at P < 0.05.

## 3 Results

Out of 920 HCWs (i.e., clinical, administrative, and cleaning staff), 140 workers agreed to participate in this study (participation rate = 15.2%). The majority of participants were female employees (67.9%) and married (73.6%). The mean age and work experience of participants were 34.24 (7.31) and 8.77 (7.52) years, respectively. In addition, the majority of the participants had less than five years of work experience (45.7%). The mean weekly working hours of the participants were 38.96 (7.28) hours. Twenty percent of the participants had a history of being infected with COVID-19, and 24.3% of them had a history of being quarantined due to COVID-19. Other personal characteristics of the participants are presented in Table 1.

**Table 1:** Demographic characteristics of the participants (N = 140).

Variable	N	(%)
**Sex**		
Male	45	32.1
Female	95	67.9
**Marital status**		
Single	37	26.5
Married	103	73.5
**Types of employees**		
Clinical staffs	80	57.1
Administrative staffs	21	15.0
Cleaning staffs	39	27.9
**Education**		
Diploma	38	27.1
Bachelor's degree	88	62.9
Master’s degree and above	14	10.0
**Work shift**		
Fixed	24	17.1
Rotated	116	82.9
**Work unit**		
General	100	71.4
Intensive care	40	28.6
**Having a history of being infected with COVID-19**	29	20.7
**Having a history of quarantine**	34	24.3
	**Mean**	**SD**
**Age (year)**	34.24	7.31
**Work experience (year)**	8.77	7.52
**Working hours per week**	38.96	7.28
**Work in the COVID-19 pandemic (day)**	108.09	73.23

*Note*. COVID-19: Coronavirus disease 19; SD: Standard deviation.

In the present study, 22.9% (n = 32) of participants reported GAD while the mean scores of anxiety and depression were 6.64 (4.86) and 18.21 (10.59), respectively. Nineteen percent (n = 27) and 38.6% (n = 54) of them experienced mild to moderate and severe depression, respectively.

Table 2 provides the multivariable linear regression analysis to examine the relationship among anxiety, depression, and demographic characteristics of HCWs in which anxiety was defined as an outcome variable. A direct significant association was observed between the anxiety and depression (b = 0.32, 95% CI = 0.27, 0.37), being female (b = 1.63, 95% CI = 0.30, 2.69), and having a history of being infected with COVID-19 (b = 2.29, 95% CI = 0.95, 3.64).

**Table 2:** Association between anxiety and depression and demographic characteristics of healthcare workers (N = 140).

Variable	b^a^	SE^b^	95% CI^c^	P-value
Depression	0.32	0.27	0.27, 0.37	<0.001
Age	0.02	0.09	-0.17, 0.21	0.831
Female (ref^d^ to male)	1.63	0.67	0.30, 2.96	0.016
Being single (ref^d^ to being married)	-0.61	0.66	-1.91, 0.67	0.35
Educational level (ref^d^ to diploma)				
Bachelor's degree	0.41	0.87	-1.29, 2.12	0.63
Master's degree and above	-.1.28	1.25	-3.70, 1.12	0.29
Types of employees (ref^d^ to clinical staff)				
Administrative staff	1.69	0.98	-0.23, 3.61	0.08
Cleaning staff	1.10	1.00	-0.86, 3.06	0.27
Rotated work shift (ref^d^ to fixed work shift)	1.36	0.81	-0.23, 2.95	0.09
Work experience	0.04	0.10	-0.15, 0.24	0.69
Working hours per week	0.02	0.04	-0.53, 0.10	0.51
Work in the COVID-19 pandemic (day)	0.07	0.63	-1.16, 1.32	0.90
Having a history of being quarantine	0.29	0.68	-1.04, 1.63	0.66
Having a history of being infected with COVID-19	2.29	0.68	0.95, 3.64	0.001
Intensive care unit (ref^d^ to general)	0.05	0.66	-1.24, 1.34	0.08

*Note*. COVID-19: Coronavirus disease 19.

a b coefficient was obtained according to the multivariable linear regression.

b Standard error.

c Confidence interval.

d Reference group.

Table 3 presents the multivariable linear regression analysis to investigate the relationship between depression and anxiety and demographic characteristics of HCWs where depression was defined as an outcome variable. As shown, a direct association was found between depression and anxiety (b = 1.55, 95% CI = 1.30, 1.81) and having a history of being quarantined due to COVID-19 (b = 3.16, 95% CI = 0.26, 6.05).

**Table 3:** Association between depression and anxiety and demographic characteristics of healthcare workers (N = 140).

Variable	b^a^	SE^b^	95% CI^c^	P-value
**Anxiety**	1.55	0.13	1.30, 1.81	<0.001
**Age**	0.18	0.21	-0.24, 0.61	0.39
**Female (ref**^d^**to male**)	-0.19	1.52	-3.17, 2.79	0.90
**Being single (ref**^d^**to being married**)	0.83	1.45	-2.02, 3.69	0.57
**Educational level (ref**^d^**to diploma**)				
Bachelor's degree	-1.88	1.90	-5.62, 1.85	0.32
Master's degree and above	1.47	2.71	-3.84, 6.79	0.58
**Types Of employees (ref**^d^**to clinical staff**)				
Administrative staffs	-3.76	2.15	-8.00, 0.46	0.32
Cleaning staffs	-2.03	2.20	-6.35, 2.28	0.58
**Rotated work shift (ref**^d^**to fixed**)	-2.28	1.79	-5.79, 1.23	0.20
**Work experience**	-0.32	0.22	-0.75, 0.11	0.14
**Working hours per week**	-0.13	0.08	-0.31, 0.03	0.12
**Work in the COVID-19 pandemic (day)**	-0.49	1.39	-3.21, 2.23	0.72
**Having a history of being quarantine**	3.16	1.47	0.26, 6.05	0.03
**Having a history of being infected with COVID-19**	3.06	1.54	-0.05, 6.07	0.06
**Intensive care unit (ref**^d^**to general**)				

*Note*. COVID-19: Coronavirus disease 19.

a b coefficient was obtained according to the multivariable linear regression.

b Standard error.

c Confidence interval.

d Reference group.

## 4 Discussion

This study was conducted to determine the anxiety and depression levels among HCWs during the COVID-19 pandemic and investigate the association between them regarding their demographic characteristics. According to the obtained results, HCWs experienced some degree of anxiety and depression during the outbreak. In addition, a direct relationship was reported between anxiety and depression levels.

According to the results, the mean anxiety score of HCWs was 6.64, and approximately 23% of participants reported GAD. Similarly, surveying the psychological distress among healthcare providers during the COVID-19 crisis, Margaretha et al. found that 33.3% of participants experienced anxiety [30]. A systematic review has evidenced that 23.2% of HCWs experienced anxiety symptoms during the COVID-19 pandemic [31]. However, an online-based study in Egypt demonstrated that 90.5% of HCWs who faced the COVID-19 pandemic experienced different degrees of anxiety [32]. The reason for such inconsistency can be attributed to differences in the samples and working conditions in the two studies. In the study conducted in Egypt, the sample size was 316, and more than 70% of participants were physicians and nurses. In addition, according to a report by the World Bank, Egypt is facing a shortage of physicians and nurses, and this statistics is 0.5 per 1000 people for physicians and 1.9 per 1000 for nurses [32]. Therefore, the shortage of staff led to a high workload [33, 34], resulting in increased anxiety symptoms during the COVID-19 pandemic [35].

Anxiety among HCWs during the COVID-19 pandemic may be related to the shortage of personal protective equipment and other necessary equipment in the early phases of the epidemic [36]. Likewise, one of the important challenges of the Iranian healthcare system in the early months of the COVID-19 pandemic was the shortage of personal protective equipment [37]. This shortage seems to have affected the level of anxiety reported in the current study. Therefore, the provision of adequate staff and personal protective equipment may protect HCWs against the psychological adverse effects of the COVID-19 pandemic.

In the present study, 57.9% of participants experienced some degree of depression. According to a systematic review report, 22.8% of HCWs had symptoms of depression during the COVID-19 [31]. In their study, Aly et al. reported that 94% of healthcare providers experienced mild to severe depression during the COVID-19 pandemic [32]. Long working hours have been identified as a risk factor for anxiety and depression [38]. Therefore, long working hours in these exhausting conditions [39] can be recognized as the reason for the higher prevalence of depression and anxiety in the above-mentioned study [32].

In the present study, a direct significant relationship was observed between anxiety and depression. Anxiety and depression are two different mental disorders, but due to the common pathophysiology of the diseases, including changes in serotonin and norepinephrine transport, the possibility of both occurring at the same time is expected [40]. Furthermore, the relationship between depression and anxiety has been confirmed in previous studies. For instance, a significant direct association was identified between depression and anxiety levels among Italian HCWs [41]. In addition, during the outbreak of diseases such as SARS or Ebola, HCWs were at greater risk of anxiety and depression at the same time [42].

According to the findings of the present study, the female gender among HCWs was associated with higher anxiety levels. It has been reported by other studies in Iran that female HCWs and the female general population experienced more anxiety during the COVID-19 pandemic [43, 44]. Females working in medical centers are uniquely affected by various stressors including role pressure, difficulty in balancing work life, and lack of adequate support; therefore, it is expected that their psychological distress increase during the COVID-19 pandemic [24]. In addition, previous researches have demonstrated that women are more sensitive, fragile, more vulnerable to anxiety than men. For instance, in a study conducted during the SARS outbreak, females were more likely to seek emotional counseling than men [45].

Our study detected a significant relationship between having a history of infection with COVID-19 and anxiety level among the HCWs. A previous study reported that the prevalence of anxiety symptoms was higher in the individuals infected with the COVID-19 [43]. In addition, another study in the general population revealed that people who were quarantined due to being infected with COVID-19 experienced higher anxiety levels than healthy people [46]. It seems that employees experienced more anxiety when they were afflicted with the disease or had a fear that their family members and friends, who are in contact with them, get infected with the virus.

Furthermore, there was a direct relationship between having a history of being quarantined and higher depression symptoms. A longitudinal mediation analysis showed that lack of social connection was associated with social isolation and significantly higher levels of depression [47]. In addition, HCWs observe the patient's suffering and death, have anxiety due to close contact with patients, experience long-term separation from family members that can affect their mental health [48]. Uncertainty, quarantine completion length, separation from the beloved people, and the lack of support lead to feelings of loneliness, but for cutting the transmission chain of COVID-19 disease, there is no choice but to stay away from the situation. Non-pharmacological interventions can have a positive effect on the psychological effects of illness and quarantine. For instance, it has been indicated that non-pharmacological techniques such as yoga, music therapy, and aromatherapy can be used to overcome anxiety and depression [49, 50].

Pandemic is a substantial stressor for a country's political, economic, and health system. Based on the WHO report, mental health services were restricted during the COVID-19 period in 93% of countries worldwide; however, there is an urgent need for access to these services due to the devastating effects of this disease on mental health [51,52]. Since resources could be scarce in serious pandemic circumstances, appropriate and timely psychological support can be applied in many forms such as telemedicine groups and informal support [53].

### 4.1 Limitations of the study

The present study has some limitations. First, due to the pandemic's sudden onset, it was impossible to assess and adjust baseline individuals' anxiety and depression conditions as covariates. Hence, the obtained information should not be compared with non-crisis situations. In addition, the participation rate of the HCWs in this study was low, and this may lead to a negative bias. Indeed, it is possible that people who were more socially engaged had participated in the study, thus they might not be representative of the study population [54]. Moreover, the socioeconomic status could vary in the study participants' age range and associate with anxiety and depression, which was not assessed in the present study. Finally, the small number of participants limited the possibility of using statistical models.

## 5 Conclusion

Based on the findings of the present research and other similar studies, in the face of critical situations such as the outbreak of COVID-19, HCWs may suffer from varying degrees of psychological disorders. According to our results, some socio-demographic variables such as gender and a history of infection with COVID-19 affect the level of anxiety. Additionally, having a history of being quarantined has a direct correlation with depression. Given the key role of HCWs in providing high-quality care for patients, attention to their mental health should be a priority for health system planners and policymakers. Eventually, appropriate interventions must be taken to improve their psychological condition, especially in serious pandemic situations.

## Declarations

### Author contribution statement

Saeideh Motahedi, Mahboobeh Khajeh: Conceived and designed the experiments; Performed the experiments; Wrote the paper.

Nasrin Fadaee Aghdam, Robabe Baha, Abbas Mardani: Analyzed and interpreted the data; Contributed reagents, materials, analysis tools or data; Wrote the paper.

Hossein Bagheri: Conceived and designed the experiments; Wrote the paper.

Roqayeh Aliyari: Analyzed and interpreted the data; Wrote the paper.

### Funding statement

This research did not receive any specific grant from funding agencies in the public, commercial, or not-for-profit sectors.

### Data availability statement

Data will be made available on request.

### Declaration of interests statement

The authors declare no conflict of interest.

### Additional information

No additional information is available for this paper.

## Figures and Tables

**Table:** 

**Demographic Questionnaire**

1. Age (years): ...
2. Gender: Male ☐ Female ☐
3. Marital status: Single ☐ Married ☐
4. Occupation: Clinical staffs ☐ Administrative staffs ☐ Cleaning staffs ☐
5. Education level: Diploma ☐ Bachelor ☐ Master ☐ Doctorate ☐
6. Work experience (year): …
7. Shift type: Fixed ☐ Rotating ☐
8. How long do you work in the COVID-19 pandemic (day): …
9. How many hours a week do you work in the hospital (hours)? …
10. Are you infected with COVID-19? Yes ☐ No☐
11. Have you been quarantined due to COVID-19? Yes ☐ No☐
12. Which department do you work in? General unit ☐ Critical unit ☐

**Table:** 

Over the last 2 weeks, how often have you been bothered by the following problems?	Not at all sure	Several days	Over half the days	Nearly every day
1. Feeling nervous, anxious, or on edge	0	1	2	3
2. Not being able to stop or control worrying	0	1	2	3
3. Worrying too much about different things	0	1	2	3
4. Trouble relaxing	0	1	2	3
5. Being so restless that it’s hard to sit still	0	1	2	3
6. Becoming easily annoyed or irritable	0	1	2	3
7. Feeling afraid as if something awful might happen *Add the score for each column* Total Score *(add your column scores)* =	0	1	2	3
	+	+	+	
			

**Table:** 

Below is a list of the ways you might have felt or behaved. Please tell me how often you have felt this way during the past week.	During the Past Week			
	Rarely or none of the time (less than 1 day)	Some or a little of the time (1–2 days)	Occasionally or a moderate amount of time (3–4 days)	Most or all of the time (5–7 days)
1. I was bothered by things that usually don't bother me.	☐	☐	☐	☐
2. I did not feel like eating; my appetite was poor.	☐	☐	☐	☐
3. I felt that I could not shake off the blues even with help from my family or friends.	☐	☐	☐	☐
4. I felt I was just as good as other people.	☐	☐	☐	☐
5. I had trouble keeping my mind on what I was doing.	☐	☐	☐	☐
6. I felt depressed.	☐	☐	☐	☐
7. I felt that everything I did was an effort.	☐	☐	☐	☐
8. I felt hopeful about the future.	☐	☐	☐	☐
9. I thought my life had been a failure.	☐	☐	☐	☐
10. I felt fearful.	☐	☐	☐	☐
11. My sleep was restless.	☐	☐	☐	☐
12. I was happy.	☐	☐	☐	☐
13. I talked less than usual.	☐	☐	☐	☐
14. I felt lonely.	☐	☐	☐	☐
15. People were unfriendly.	☐	☐	☐	☐
16. I enjoyed life.	☐	☐	☐	☐
17. I had crying spells.	☐	☐	☐	☐
18. I felt sad.	☐	☐	☐	☐
19. I felt that people dislike me.	☐	☐	☐	☐
20. I could not get “going.”	☐	☐	☐	☐

SCORING: 0, 1, 2, and 3 for answers in the first, second, third, and fourth columns, respectively. The scoring of positive items is reversed. The possible scores are in the range of 0–60 and the higher scores indicate the presence of more symptomatology.

## Appendix

### Appendix B. Generalized Anxiety Disorder 7-item (GAD-7) Scale

Developed by Drs. Robert L. Spitzer, Janet B.W. Williams, Kurt Kroenke et al. (2006) with an educational grant from Pfizer Inc. No permission required to reproduce, translate, display, or distribute.
